# Dysregulation of mTORC1/autophagy axis in senescence

**DOI:** 10.18632/aging.101277

**Published:** 2017-08-05

**Authors:** Bernadette Carroll, Viktor I. Korolchuk

**Affiliations:** Institute for Cell and Molecular Biosciences; Newcastle University; Newcastle upon Tyne NE4 5PL, UK

**Keywords:** senescence, mTORC1, autophagy

Cellular senescence is a potent tumour suppressor mechanism whereby cells irreversibly exit from the cell cycle in response to excessive cellular stress such as DNA damage or oncogene activation. Senescence thus prevents uncontrolled proliferation and tumourigenesis. Senescent cells however maintain a pro-growth pheno-type which in the absence of proliferation drives increased cell size, increased organelle content (including mitochondria and lysosomes) and secretion of pro-inflammatory signals [1, 2]. The mechanisms underlying these pro-growth phenotypes are not well understood but activation of the mammalian target of rapamycin complex 1 (mTORC1) has long been associated with senescence [2-4]. Indeed, the mTORC1 inhibitor rapamycin can, depending on the timing of the intervention, inhibit, delay or dampen senescence-associated phenotypes such as cell size and secretion of inflammatory cytokines.

Both mTORC1 and senescence are intimately linked to aging and age-related phenotypes [1, 3]. The burden of senescent cells increases *in vivo* with age and the highly inflammatory nature of these cells may negatively impact the surrounding cells and tissues. Clearance of p16-positive (senescent) cells improves both health- and life-span in rodent models [5] while rapamycin is a robust mediator of lifespan extension across an evolutionary diverse range of organisms [1, 3]. Therefore understanding how this important tumour suppressor mechanism works is an extremely important question for gerontologists and cancer biologists alike.

Despite the central role for mTORC1 activity in cell senescence, the mechanisms controlling mTORC1 and how it supports the strong pro-growth phenotype are unknown. In proliferating cells, mitogenic signals including growth factors, energy and amino acid availability tightly regulate the activity of mTORC1 to balance anabolic (protein translation, nucleotide synthesis) and catabolic (autophagy) processes [6]. We demonstrated however that the acquisition of senescence is associated with a rewiring of signalling pathways that renders mTORC1 significantly less sensitive to nutrient availability [7]. mTORC1 remains active even in the complete absence of any growth factors or amino acids and prevents starvation-induced activation of autophagy. It remains to be seen however whether mTORC1 remains responsive to other mitogenic inputs such as energy and oxygen availability.

We have only just begun to unravel the complex rewiring that occurs during senescence to drive this insensitivity but evidence suggests that changes in both growth factor pathways and autophagy-dependent amino acid availability contribute. Firstly, growth factor signalling through the PI3K/Akt pathway persists in starved senescent cells. This is driven, via poorly understood mechanisms, by hypopolarisation of the plasma membrane which causes defects in primary cilia formation. As a result, the mTORC1 negative regulator, TSC2 is not robustly recruited to lysosomes and mTORC1 remains active. Interestingly, hypopolarisa-tion of the plasma membrane directly contributes to the acquisition of senescence since long term treatment to maintain plasma membrane potential delays the onset of replicative senescence. It's not clear how this reduced membrane potential occurs, for example whether it is simply due to increased cell size (and inability to produce enough membrane, channels or electrolytes), or reduced energy, or imbalance of NADP(H)/ROS levels. How hypopolarisation of the plasma membrane subsequently leads to a failure of cilia growth and persistent signalling through PI3K/Akt pathway, is also not clear. Growth factor defects downstream of cilia have been reported previously [8]. One hypothesis is that in the absence of cilia, specific phosphatases or other regulators fail to localise properly to the membrane and thus PI3K/Akt signalling persists.

Secondly, autophagy appears to play a complex and somewhat contradictory role in senescence, similar to that seen in many cancers. Senescent cells have elevated levels of autophagy in basal conditions and we demonstrated that this elevated autophagy supports mTORC1 activity and maintains more mTORC1 at lysosomes compared to control cells. At the same time, persistent mTORC1 signalling restrains autophagy to non-toxic levels. Inhibition of mTORC1 or perturbation of autophagy leads to senescent cell death indicating that they rely on mTORC1 and autophagy for their survival in stress conditions. More studies are required to tease out the exact mechanisms of this cell death and it will be interesting to try and mechanistically uncouple or directly link mTORC1 and autophagy defects in senescence.

Understanding the basic mechanisms driving cellular aging is an increasingly important question in biomedicine today. Interventions to improve healthspan could have far reaching societal and economic impacts. Insights from cell senescence are equally important to those studying basic mechanisms of cell growth, aging and cancer biologists. In addition, our findings suggest that mechanisms described in senescence could also be of interest in the study of ciliopathies. While we continue to work out the basic mechanism driving and supporting senescence, one of the most acute questions that remains to be answered is whether coupling dietary interventions and direct targeting of mTORC1/autophagy can be exploited to promote the clearance of senescent cells *in vivo*.
